# Genomic sequencing identifies tuberculosis cluster in inner-city Sydney boarding house, Australia, 2022

**DOI:** 10.5365/wpsar.2025.16.4.1153

**Published:** 2025-12-03

**Authors:** Eunice Stiboy, Standish Rigava, Anthea Katelaris, Vicky Sheppeard, Anna Glynn-Robinson, Yasmeen Al-Hindawi, Hazel Goldberg, Kerrie Shaw, Vitali Sintchenko, Elena Martinez, Taryn Crighton, Ellen Donnan, Anthony Byrne

**Affiliations:** a South Eastern Sydney Public Health Unit, New South Wales Health, Sydney, New South Wales, Australia.; b National Centre for Epidemiology and Population Health, Australian National University, Canberra, Australian Capital Territory, Australia.; c St Vincent's Health Network Sydney, Sydney, New South Wales, Australia.; d School of Public Health, University of Sydney, Sydney, New South Wales, Australia.; e Consultant in Respiratory and Tuberculosis Medicine, Prince of Wales Hospital, Sydney, New South Wales, Australia.; f Sydney Eye Hospital and Royal Prince Alfred Hospital, Sydney, New South Wales, Australia.; g South Eastern Sydney Local Health District, New South Wales Health, Sydney, New South Wales, Australia.; h New South Wales Mycobacterium Reference Laboratory, Institute of Clinical Pathology and Medical Research, New South Wales Health Pathology, Westmead, New South Wales, Australia.; i Sydney Infectious Diseases Institute and School of Medical Sciences, The University of Sydney, Sydney, New South Wales, Australia.; j New South Wales Tuberculosis Program, Communicable Diseases Branch, Health Protection New South Wales, Sydney, New South Wales, Australia.; k University of New South Wales, Sydney, New South Wales, Australia.

## Abstract

**Objective:**

In 2022, the New South Wales TB Program was notified of genomically clustered *Mycobacterium tuberculosis* isolates from two smear-positive tuberculosis (TB) patients diagnosed 3 months apart. Secondary investigations found they resided in the same Sydney boarding house. The objective of this study was to investigate this cluster and conduct active case finding among contacts.

**Methods:**

We conducted a site visit to understand transmission risk, reviewed patient histories, performed a risk assessment and conducted on-site TB contact screening, including interferon-gamma release assay testing. Long-term residents were also screened via chest X-ray. Past residents were referred to local TB services.

**Results:**

Four residents with TB disease were identified, three of whom were genomically linked to the cluster. The exposure period in the boarding house was determined to be from January 2021 to September 2022. All residents and staff were considered contacts requiring screening. Of the 91 contacts identified, 37 (41%) completed screening, including 20 (22%) who attended the on-site clinic. Among those screened, one resident with TB disease (patient 4) and three residents and one staff member with TB infection were identified.

**Discussion:**

This cluster highlights the role of genomic sequencing in detecting TB transmission. The first three patients were infectious for prolonged periods before diagnosis, likely facilitating transmission in communal areas. In multidwelling buildings with TB exposures, contact screening of all residents may be required when prolonged exposures are found. Strategies to increase screening completion should be further explored.

Australia has one of the world’s lowest incidence rates of tuberculosis (TB) and has maintained this status through excellent TB control over the last three decades, despite its proximity to countries with some of the highest incidences in the world. (*1*) In 2018, Australia reported 1438 TB notifications, representing a rate of 5.8 per 100 000 people, with 89% of people with TB disease (active TB) reported in people who were born overseas. (*1*) In the 4 years from 2015 to 2018, the most frequently reported countries of birth for people with TB disease diagnosed in Australia were China, India, Nepal, the Philippines and Viet Nam. (*1*)

In 2022, the rate of TB in New South Wales (NSW) was 6.5 per 100 000 people, with these notifications comprising 41% of the total notifications in Australia. (*2*) The majority (*n* = 481, 91%) of the 528 notified people with TB disease were reported as having been born overseas, with the most frequently reported countries of birth being India and Nepal. (*2*) Notification rates by country of birth ranged from 104.7 per 100 000 people for those born in Nepal to 0.9 per 100 000 for those born in Australia. (*2*)

During 2016, NSW Health introduced routine whole genome sequencing (WGS) for all *Mycobacterium tuberculosis* isolates, which has allowed for the surveillance of circulating strains, detection of genotypic drug susceptibility, differentiation of TB endogenous relapse from exogenous reinfection, inference of transmission pathways and cluster detection to guide public health responses, as well as detection of laboratory cross-contamination. (*3*, *4*) Routine WGS has the potential to allow for better targeting of contact tracing interventions and prevention strategies. (*4*)

With Australia being a country of low incidence for TB, transmission among the general population is expected to be uncommon, with most incident TB generated by reactivation of TB infection (latent TB infection) acquired overseas. (*5*, *6*) Local transmission is occasionally recognized among households, but less commonly in health-care facilities or congregate settings such as prisons or facilities for people experiencing homelessness. (*6*) This can result in delayed identification of transmission, which may have a significant epidemiological and public health impact. (*5*) A comprehensive understanding of local epidemiology and transmission is crucial in low-incidence countries, as detailed by the World Health Organization’s *Framework towards TB elimination in low-incidence countries*. (*6*) The Framework outlines the requirement for tailored approaches to contain local outbreaks, with rigorous contact investigations and outbreak management being essential elements for control of TB in low-incidence settings. (*6*)

We report a cluster of TB cases in a boarding house in inner-city Sydney, NSW, which was detected via routine WGS.

## Methods

In November 2022, the South Eastern Sydney Local Health District’s Public Health Unit (PHU) and the chest clinic at St Vincent’s Hospital (SVH) were notified by the NSW Health TB Program of genomically clustered *M. tuberculosis* isolates from two TB patients with a single nucleotide polymorphism (SNP) difference on routine WGS. At the time of diagnosis of these patients (3 months apart), it was apparent that they shared the same residential address, a boarding house in inner-city Sydney, but were not known to one another or identified during individual patient follow-up when prompted about boarding house exposures.

### Epidemiological investigation

Epidemiological investigation was undertaken by the SVH chest clinic in accordance with the NSW TB control and contact management guidelines. (*7*, *8*) Each patient was interviewed at the time of diagnosis, and a risk assessment and follow-up of disclosed contacts were undertaken. Following WGS results, the boarding house was contacted and a list of residents who stayed in the boarding house during the exposure period was obtained. An inspection of the boarding house was undertaken to assess transmission risk. The boarding house manager was interviewed to determine the movements of staff and residents within the building.

### Boarding house screening

During the preliminary investigation, one of the patients reported that a relative who had previously lived with them in the boarding house had been diagnosed with pulmonary TB after leaving Australia. Investigations into this patient found their symptom onset was approximately April–May 2021, so the potential risk period was updated to begin in January 2021 (to include a margin of uncertainty).

We conducted a risk assessment to determine the screening strategy. We established that all boarding house residents, staff and visitors (who resided there for ≥ 7 days) warranted contact tracing and screening for TB infection for the period from January 2021 to September 2022 (when the second patient was admitted to hospital).

### Laboratory methods

WGS was performed on all culture-positive isolates by the Mycobacterium Reference Laboratory NSW. Briefly, DNA was extracted from pure cultures by an in-house method, and a library constructed using Nextera XT (Illumina, San Diego, CA, United States of America). Sequencing was performed using NextSeq 500 (Illumina) with a 150-bp paired-end protocol using NextSeq 500/550 v2 kits. Analysis was performed as described by Lam et al., after samples passed quality control and were mapped against the reference genome of *M. tuberculosis* H37RV. (*3*) Clustering was defined as isolates having ≤ 12 SNPs.

### Contact tracing methods

Identified contacts were screened for TB infection via a single interferon-gamma release assay (IGRA), as it was more than 8 weeks since last exposure to an infectious patient. Residents identified as long-term (defined as living in the boarding house for > 2 years) were considered higher risk and offered a chest X-ray (CXR) in addition to IGRA testing. (*9*, *10*)

Residents and staff of the boarding house were offered screening at a one-off clinic held at the boarding house. History of TB symptoms, previous TB exposures, TB vaccination, and demographic details, including country of birth and duration of residence or work at the boarding house, were collected. Blood for IGRA testing was collected on-site, and long-term residents were given a referral for a CXR that could be conducted nearby. Current residents and staff who did not attend the on-site clinic were referred to the local chest clinic.

Past residents and staff were contacted by phone and notified of the potential TB risk. After their TB risk history was obtained, they were referred to their nearest chest clinic for screening and follow-up. They were also sent written information on exposure. The NSW TB Program provided support by facilitating the use of a call centre initially set up for COVID-19 contact tracing. Attempts to contact past residents were made in two rounds of calls on different dates, with up to three calls per round. Contact attempts were made via e-mail, if available. Contacts in other health districts in NSW were referred to their local chest clinic, and those living in other states or territories were notified to their jurisdictional TB programme for follow-up.

## Results

Including the three people with TB disease identified at the start of the investigations, we identified 94 people living or working in the boarding house during the period of interest from January 2021 to September 2022. No visitors were identified during the exposure period. During the contact investigations, a fourth resident with TB disease (patient 4) was diagnosed during a routine medical exam for an unrelated health condition, bringing the total number of TB patients in the building to four (4%) of 94 people. A summary of the cohort can be found in **Table 1**.

**Table 1:** Summary of TB screening results for all residents and staff (*n* = 94) of a boarding house, Sydney, NSW, Australia, 2022

-	Current residents	Past residents	Staff	Total
**TB disease**	**3**	**1**	**0**	**4**
**Positive IGRA**	**3**	**0**	**1**	**4**
**Negative screening**	**17**	**14**	**1**	**32**
**Not screened – unable to contact**	**0**	**22**	**0**	**22**
**Not screened – contacted but declined screening**	**7**	**1**	**3**	**11**
**Not screened – contacted but did not attend or lost ** **to follow-up**	**5**	**16**	**0**	**21**
**Total**	**35**	**54**	**5**	**94**

IGRA: interferon gamma release assay; NSW: New South Wales; TB: tuberculosis.

### TB disease patients

We identified four people with fully susceptible TB disease in this cluster. Their demographic and clinical characteristics are summarized in **Table 2**.

**Table 2:** Summary of patients with TB disease and contacts with positive IGRA who were identified during contact screening of residents of a boarding house, Sydney, NSW, Australia, 2022

Patients with TB disease				
	Patient 1	Patient 2	Patient 3	Patient 4
**Sex**	**Female**	**Female**	**Male**	**Male**
**Onset**	**April 2022**	**August 2022**	**~May 2021**	**Asymptomatic**
**Symptoms**	**Cough, 10 weeks** **Fever** **Night sweats** **Weight loss**	**Cough, unknown duration** **Iron deficiency** **Weight loss** **Nausea, vomiting**	**Undefined respiratory symptoms**	**Asymptomatic**
**Laboratory and imaging results**	**Sputum microscopy (3+) and culture-positive for AFB** **Cavities on CXR**	**Sputum microscopy (2+) and culture-positive for AFB** **Cavities on CXR**	**Reported to have cavities on imaging**	**Bronchoscopy microscopy, PCR and culture positive for AFB** **Subtle changes on routine CT scan**
**Diagnosis**	**July 2022**	**October 2022**	**~August 2021 (overseas)**	**December 2022**
**Risk factors**	**Childhood exposure to TB**	**Diabetes**	**Childhood exposure to TB**	**COPD** **Previous unrelated TB exposure, 2019a**
**Country of birth**	**Low-incidence country**	**Low-incidence country**	**Low-incidence country**	**Australia**
**Screened contacts with positive IGRA**				
	**Contact 1**	**Contact 2**	**Contact 3**	**Contact 4**
**Sex**	**Male**	**Male**	**Female**	**Male**
**Arrival in boarding house**	**April 2016**	**March 2014**	**March 2017**	**Staff, lives off-site**
**Symptoms**	**Asymptomatic**	**Asymptomatic**	**Asymptomatic**	**Asymptomatic**
**Laboratory results**	**IGRA-positive** **CXR-negative**	**IGRA-positive** **CXR-negative**	**IGRA-positive** **CXR-negative**	**IGRA-positive** **CXR-negative**
**Diagnosis**	**December 2022**	**December 2022**	**December 2022**	**December 2022**
**Previous risk factors**	**N/A**	**N/A**	**N/A**	**Born and lived in high-incidence country**
**Country of birth**	**Australia**	**Australia**	**Australia**	**High-incidence country**

AFB: acid-fast bacilli; COPD: chronic obstructive pulmonary disorder; CT: computer tomography; CXR: chest X-ray; IGRA: interferon gamma release assay; NSW: New South Wales; N/A: not applicable; PCR: polymerase chain reaction; TB: tuberculosis.

^a^ TB infection treated.

Patient 1, a female in her 20s, presented to the hospital emergency department in July 2022, with a 4-month history of weakness, weight loss, fever, chills and non-productive cough. Sputum testing was both culture- and smear-positive for TB. She reported that increasing breathlessness and cough meant she had spent considerable time using the shared stairwell to reach her room. While she did report TB exposure during childhood, we considered this diagnosis to be from a more recent exposure to her relative, patient 3 (below). During her initial interview, she confirmed her residential address to be the boarding house, but no high-risk contacts were identified (as defined in the NSW TB contact investigation guidelines). (*8*) As she reported no contact with other residents apart from her relative (patient 3) and did not spend time in the communal areas with other residents, no additional screening was undertaken at the facility.

Patient 2, a female in her 50s, was admitted to hospital in September 2022 with abdominal pain, a 6-week history of weight loss, nausea and vomiting, iron deficiency and a cough of unknown duration. Sputum testing was both culture- and smear-positive for TB. The SVH chest clinic noted the same residential address as patient 1 on diagnosis, but no high-risk contacts were identified or additional screening undertaken at the boarding house at the time of diagnosis, as patient 2 also reported no contact with other residents. Neither patient 1 nor 2 identified one another when initial contact tracing was undertaken.

**Fig. 1:**
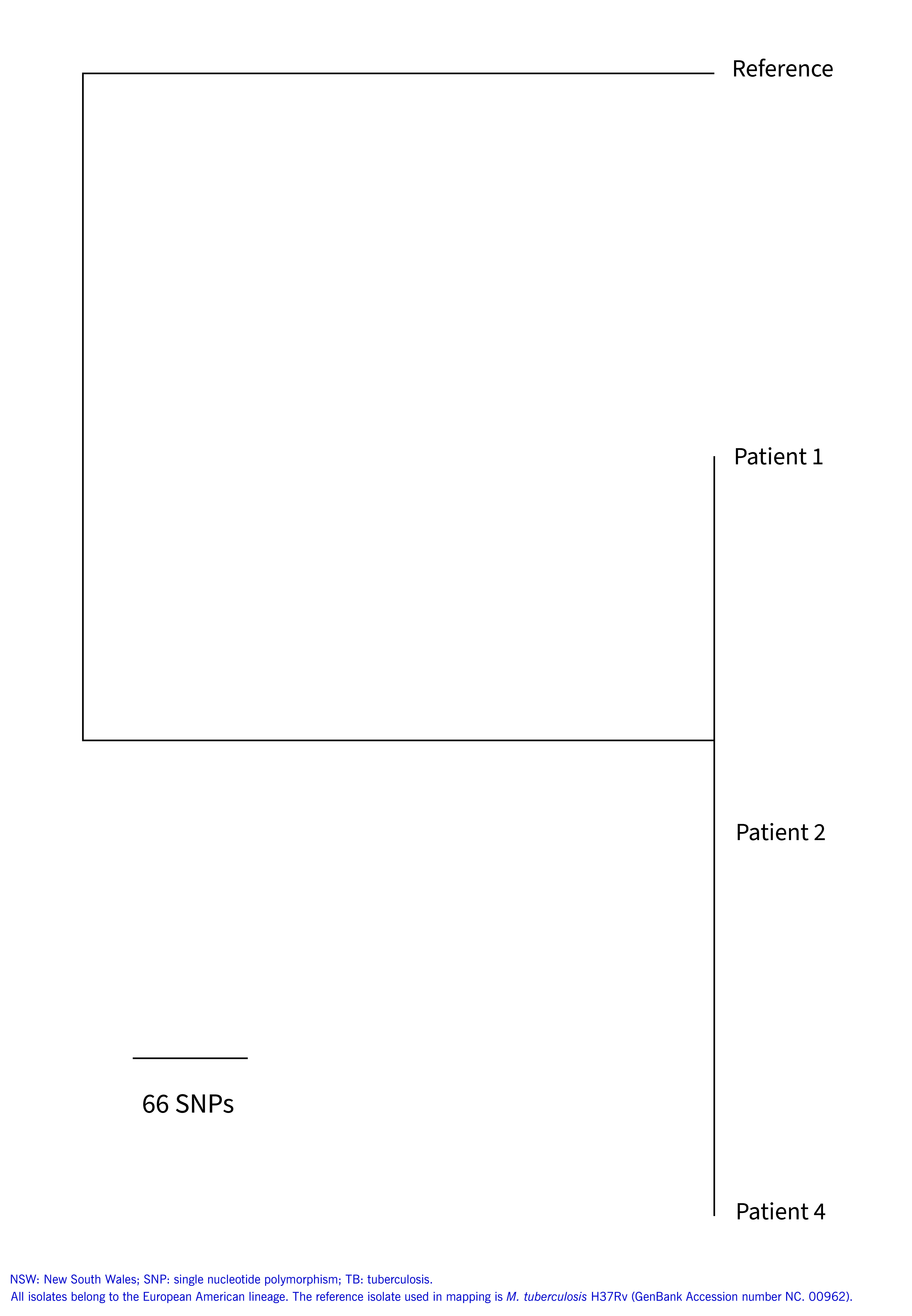
Maximum likelihood phylogenetic tree of M. tuberculosis genomes sequenced from three patients in a TB cluster in a boarding house, Sydney, NSW, Australia, 2022

In the weeks following the diagnosis of patient 2, *M. tuberculosis* isolates from patients 1 and 2 were found to be very closely related. WGS results indicated a single SNP difference between *M. tuberculosis* isolates from patients 1, 2 and 4. An isolate from patient 3 was not available for genomic sequencing. A phylogenetic tree summarizing the genomic sequencing is shown in **Fig. 1**.

[**insert** Figure 1]

The genomic sequencing results of isolates from patients 1 and 2 triggered the cluster investigation. Both patients were re-interviewed, and patient 1 reported that her relative, patient 3, who had previously lived in the boarding house, had returned to their home country 11 months earlier in August 2021, where he was diagnosed with TB disease.

Patient 3, a male in his 20s, was diagnosed with TB outside of Australia. Patient 1 reported that the onset of her relative’s cough was approximately April or May 2021 and that a CXR in their home country showed cavities. Patient 3 lived in the boarding house from January 2020 to August 2021. We consider patient 3 the likely source case in this cluster.

Patient 4 was a male in his 70s and was identified as a resident contact in the building. When routine imaging for chronic obstructive pulmonary disorder (COPD) in November 2022 showed subtle changes, he underwent further investigation. TB sputum smear and culture were negative; however, bronchoscopy specimens from December 2022 were positive for acid-fast bacilli on microscopy and culture. WGS showed his *M. tuberculosis* isolate was genomically linked to isolates from patients 1 and 2.

### Contact tracing and screening for TB infection

There were 91 residents and staff during the exposure period who required contact screening: 53 (58%) were past residents; 33 (36%) were current residents; and five (5%) were staff. There were 23 (25%) long-term residents in the cohort; duration of stay in the boarding house was unavailable for six (7%) contacts. Of the 91 residents and staff identified, 69 (76%) were contacted on-site, or by phone or e-mail. Twenty-two (24%) were uncontactable.

**Fig. 2:**
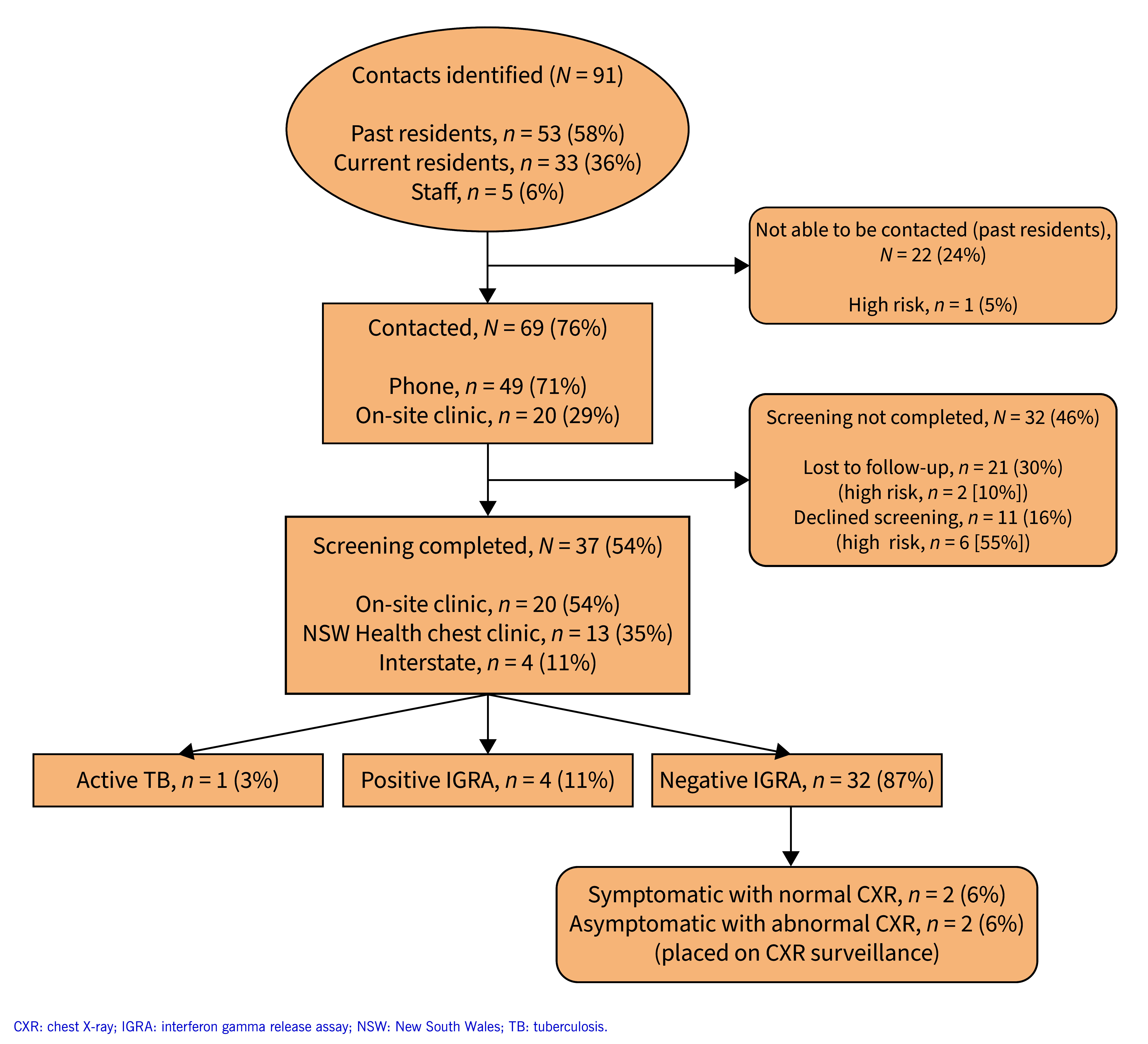
Flow chart of follow-up for the 91 contacts identified during investigation of a TB cluster in a boarding house, Sydney, NSW, Australia, 2022

Of the 69 residents and staff contacted, screening was not completed for 32 (46%) contacts: 21 (30%) were lost to follow-up, including two (10%) considered high-risk; and 11 (16%) declined to be screened, including six (55%) considered high-risk. Of the 37 (54%) contacts who completed screening, 20 (54%) attended the on-site clinic, 13 (35%) attended an NSW Health chest clinic and four (11%) were referred interstate. One (3%) screened contact was diagnosed with TB disease (patient 4). Four (11%) contacts returned a positive IGRA and 32 (87%) returned a negative IGRA. Of the 32 that returned a negative IGRA, two (6%) were symptomatic with a normal CXR and two (6%) were asymptomatic with an abnormal CXR. These four contacts were placed on CXR surveillance. A summary of the contact follow-up is shown in **Fig. 2.**

[**insert** Figure 2]

**Fig. 3:**
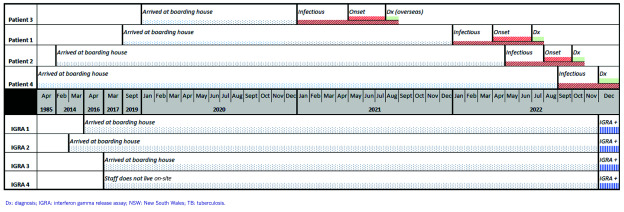
Flow chart of follow-up for the 91 contacts identified during investigation of a TB cluster in a boarding house, Sydney, NSW, Australia, 2022

The four IGRA-positive residents and staff are described in **Table 2**. Three were current long-term residents at the boarding house and were born in Australia with no known prior TB exposure or screening history. They were all asymptomatic and had normal CXRs. One reported going into the room of patient 1 on several occasions while she was unwell. The other two residents had no known direct exposure to any of the patients. The fourth IGRA-positive contact was a staff member who was born in a high-TB burden country, was asymptomatic and had a normal CXR. A timeline of the patients with TB disease and TB infection is summarized in **Fig. 3**.

[**insert** Figure 3]

### Environmental investigation results

The environmental investigation revealed that the boarding house provided both short- and long-term accommodation options and consisted of five floors including a communal rooftop. The staircase was narrow and had a landing halfway between each floor. Each floor consisted of individual studio apartments (some with private bathrooms), with shared bathrooms and toilets on each floor. The corridors were narrow and poorly ventilated. A communal laundry used by all residents was located on the rooftop. A floorplan was created based on the site visit. A review of the room history revealed that each patient with TB disease had, at some point, while infectious, resided on the same floor as a resident found to have TB infection. Patients 1 and 3 changed apartments multiple times, resulting in movement between floors.

## Discussion

We present a cluster of four patients with TB disease and four contacts with TB infection residing within the same boarding house in inner-city Sydney. Patients 1, 2 and 3 were symptomatic for long periods before diagnosis, facilitating transmission, most likely in the communal areas. The identification of this cluster has highlighted the importance and value of routine genomic sequencing of TB isolates in confirming a TB cluster in a low-incidence setting such as Australia. We have also demonstrated the value of broad contact screening when transmission has occurred in a residential building.

A major strength in the identification of this cluster was the routine application of genomic sequencing, which was able to detect genomic relatedness between patient isolates. This facilitated a prompt and targeted public health investigation and follow-up. Strong collaboration between the local chest clinic, the PHU, the NSW TB Program and pathology service, and the early formation of an outbreak management team were crucial in coordinating the cluster investigation.

The local chest clinic that conducted the patient interviews had established a working relationship with the manager at the boarding house who assisted in facilitating the distribution of risk communications to current residents and establishing the on-site clinic within a week of the investigation commencing. This ensured residents at the boarding house were provided with convenient options for screening, thus enabling our ability to promptly conduct contact follow-up and screening.

While there were many strengths in this investigation, there were also limitations. Despite intensive contact investigation efforts, we were unable to get in touch with a large proportion of the contacts identified and suspect that this may have been due to many of the former temporary residents having returned overseas. Also, we contacted a considerable number of contacts and informed them of their exposure, but they did not complete screening. This means some patients with TB infection and TB disease associated with this cluster may have been missed. Reasons why contacts did not complete screening, and whether this stemmed from health system or patient factors, remain unknown. Further work is required to understand why people do not complete TB screening if we are to increase screening completion rates and maintain a low incidence of TB in Australia. TB testing and treatment are free in public clinics in NSW, irrespective of eligibility for government-funded health care. However, we learned that a few contacts who had attempted screening were deterred from completion when they were referred to private pathology services and asked to pay a fee. In addition, some temporary residents may have been concerned that TB testing would impact immigration visas. (*11*, *12*)

Some patients were potentially exposed to several infectious patients, but we were unable to elicit the exact transmission pathways from either WGS (due to the limited SNP differences) or the patient epidemiology. One of the key questions left unanswered from this cluster was the possibility of more than one chain of transmission among the known patients. Understanding how TB was transmitted between the residents would have assisted in providing a more precise risk stratification of people in the facility. Another limitation is that we did not know whether the people with positive IGRA tests were linked to this exposure. Also, three of the four people with positive IGRA tests who had no known history of prior TB exposure cannot be ruled out, as none had received prior screening for TB infection. The fourth patient with TB infection was born in a high-burden country for TB, and we were unable to ascertain if their TB infection was a result of exposure from the boarding house or from their country of birth.

Despite the limitations of our investigation, the value of routine genomic sequencing of *M. tuberculosis* isolates for public health investigations is beneficial and is considered the gold standard for the assessment of transmission and strain relatedness. (*13*) The identification of closely related strains allows for detection of clusters that may indicate recent transmission and require more intensive public health investigation and follow-up. (*13*) The usefulness of routine WGS was demonstrated in this investigation, as clustering was only identified after genomic sequencing results were notified, which then expanded our contact and active case finding investigation. As NSW Health Pathology performs sequencing of all TB cultures, there is a possibility of detecting future cases related to this cluster, as all new sequences are compared to historical isolates. Although we were unable to determine the exact transmission pathways of this cluster, when more genomic diversity is present, genomic sequencing has the potential to suggest the direction of transmission. (*4*) Despite our investigation displaying the utility of routine genomic sequencing for TB programme development and control, results should be interpreted along with epidemiological or clinical information. (*4*, *14*)

Outbreaks of TB pose a significant risk to communities and, if left without intervention, they add to the incidence of TB disease and prevalence of TB infection, threatening the maintenance of low TB incidence and increasing the risk of local transmission. (*15*) Therefore, contact investigation and robust outbreak management are essential elements for TB control in low-incidence settings. (*16*) When local transmission is suspected, or an outbreak identified, a coordinated and prompt response based on a multidisciplinary approach is required. (*17*) A risk assessment to prioritize contact follow-up based on the infectiousness of the index patient and intensity of exposure should be considered. TB case investigation forms could be modified to include a question on type of dwelling, thus highlighting higher-risk settings at an early stage of contact identification. (*18*) This approach to the risk assessment and epidemiological investigation could help optimize resources, which are often stretched in TB programmes. (*15*)

### Conclusion

This cluster has demonstrated the value of routine genomic sequencing to identify clustering and local transmission of TB, leading to targeted public health investigation. We have shown the importance of robust outbreak investigation using a multidisciplinary team, with broad screening and multiple follow-up strategies in this boarding house environment, where residents did not necessarily know each other. Despite intensive contact tracing efforts, we could not complete screening for a substantial proportion of contacts, suggesting that methods to improve TB contact screening completeness should be explored. If countries with low TB incidence are to progress to elimination, investment in routine genomic sequencing and robust public health investigation should be commonplace in TB management guidelines and policies.
